# Resveratrol antibacterial activity against *Escherichia coli* is mediated by Z-ring formation inhibition via suppression of FtsZ expression

**DOI:** 10.1038/srep10029

**Published:** 2015-05-05

**Authors:** Dahyun Hwang, Young-Hee Lim

**Affiliations:** 1 Department of Public Health Science (BK21 PLUS program), Graduate School, Korea University; 2 Department of Laboratory Medicine, Korea University Guro Hospital, Seoul, South Korea

## Abstract

Resveratrol exhibits a potent antimicrobial activity. However, the mechanism underlying its antibacterial activity has not been shown. In this study, the antibacterial mechanism of resveratrol was investigated. To investigate induction of the SOS response, a strain containing the *lacZ*^+^gene under the control of an SOS-inducible *sulA* promoter was constructed. DNA damage was measured by pulse-field gel electrophoresis (PFGE). After resveratrol treatment, the cells were observed by confocal microscopy. For the RNA silencing assay, *ftsZ*-specific antisense peptide nucleic acid (PNA) was used. Reactive oxygen species (ROS) production increased in *Escherichia coli* after resveratrol treatment; however, cell growth was not recovered by ROS quenching, indicating that, in this experiment, ROS formation and cell death following resveratrol treatment were not directly correlated. Resveratrol treatment increased DNA fragmentation in cells, while SOS response-related gene expression levels increased in a dose-dependent manner. Cell elongation was observed after resveratrol treatment. Elongation was induced by inhibiting FtsZ, an essential cell-division protein in prokaryotes, and resulted in significant inhibition of Z-ring the formation in *E. coli*. The expression of *ftsZ* mRNA was suppressed by resveratrol. Our results indicate that resveratrol inhibits bacterial cell growth by suppressing FtsZ expression and Z-ring formation.

Antimicrobials of plant origin are effective at treating infectious diseases and because of their relatively low toxicity they also mitigate many of the side effects often associated with synthetic antibiotics^1^. An additional benefit of plant-based medicines is their lower incidence of drug-resistance than drugs from other sources^2^. The above named advantages and increasing interest in plant-derived drugs has led to active compounds such as antioxidants from plants or other natural sources being developed for a variety of purposes^3^. Flavonoids in plant extracts play a defensive role against pathogens like bacteria and fungi^4^, and plant antioxidants are now recognized as antimicrobial agents. There are many reports on the synergistic effects of antibiotics against antibiotic-resistant bacteria when combined with plant extracts^5^. For this reason, recently, many plant extracts have been investigated as alternatives to antibiotics. Resveratrol (3,4′,5-trihydroxystilbene) is a phytoalexin found in grapes, grape products, peanuts, cranberries, strawberries and some other botanical sources. It has been shown to have many beneficial effects in humans and animal models, and its antioxidative and anti-proliferative activities have promoted investigation as a chemopreventive agent for cancer and cardiovascular disease^6^,^7^,^8^,^9^,^10^,^11^. Resveratrol exhibits antimicrobial properties against bacteria, yeasts and fungi^1^. Resveratrol inhibits virulence factor expression in *Proteus mirabilis*, an important pathogen infecting urinary tract^12^ and inhibits urease activity in carcinogenic strain of *Helicobacter pylori*^13^. In addition, resveratrol has antibacterial effect on *Propionibacterium acnes*^14^, *Haemophilus ducreyi*^15^, *Arcobacter butzleri* and *Arcobacter cryaerophilus*a^16^ *and E. coli* O157:H7^17^. Recently, a report shows that reactive oxygen species (ROS) is correlated with the antibacterial activity of resveratrol^18^. Resveratrol also exhibits antiviral activity, and several mechanisms for its mode of action have been reported^19^,^20^. However, in bacteria, its precise mechanism of action has not been clearly explained. Resveratrol exhibits prooxidant activity and can induce DNA breakage in the presence of copper ions^21^,^22^,^23^. Pycnogenol, an antioxidant derived from plants, was shown to mediate the breakage of pUC19 plasmid DNA, while pre-treatment with pycnogenol was shown to protect against DNA damage induced by the Fenton reaction^24^.

ROS induce oxidative damage to DNA, thereby triggering induction of the SOS response whereby cell division is halted and DNA is repaired^25^. SulA, one of several key factors induced by the SOS response to DNA damage, has been shown to mediate inhibition of cell division in *E. coli*^26^,^27^. In bacterial cell division, FtsZ plays a key role by assembling into a contractile ring (called the Z-ring) at the midcell site of the future septum^28^. Z-ring formation is an essential step for cell division, and several proteins, including FtsZ^29^, are involved in this process. However, the exact mechanism underlying Z-ring formation is unclear. What is clear is that inhibition of Z-ring formation induces cell elongation and halts cell growth^30^. FtsZ assembly in *E. coli* is inhibited by SulA resulting in a cell division block until damaged DNA is repaired, after which SulA proteolysis restores cell division^31^.

In the present study, we investigated resveratrol’s antibacterial mechanism of action. For this purpose, we measured resveratrol-mediated ROS production, DNA fragmentation and induction of the SOS response. Additionally, we determined whether resveratrol treatment could induce cell elongation in *E. coli* and investigated the effect of resveratrol on Z-ring formation *in vivo.* To determine whether resveratrol targets FtsZ, RNA silencing using a selective RNA silencer designed to target *ftsZ* mRNA was used. Our findings confirm that FtsZ is a possible antibacterial target of resveratrol in *E. coli*.

## Results

### Antibacterial activity of resveratrol

To investigate resveratrol’s antibacterial activity we measured its MIC against *E. coli*, which was found to be 456 μg/mL. Resveratrol inhibited the growth of *E. coli* in a dose-dependent manner at concentrations equivalent to 0.125, 0.25, 0.5 and 1 MIC (μg/mL) (Figure S1). The growth of *E. coli* treated with resveratrol decreased 56.0%, 65.1%, 89.1% and 99.9% at concentrations equivalent to 0.125, 0.25, 0.5 and 1 MIC (μg/mL), respectively.

### Resveratrol-induced ROS production

**Figure 1:**
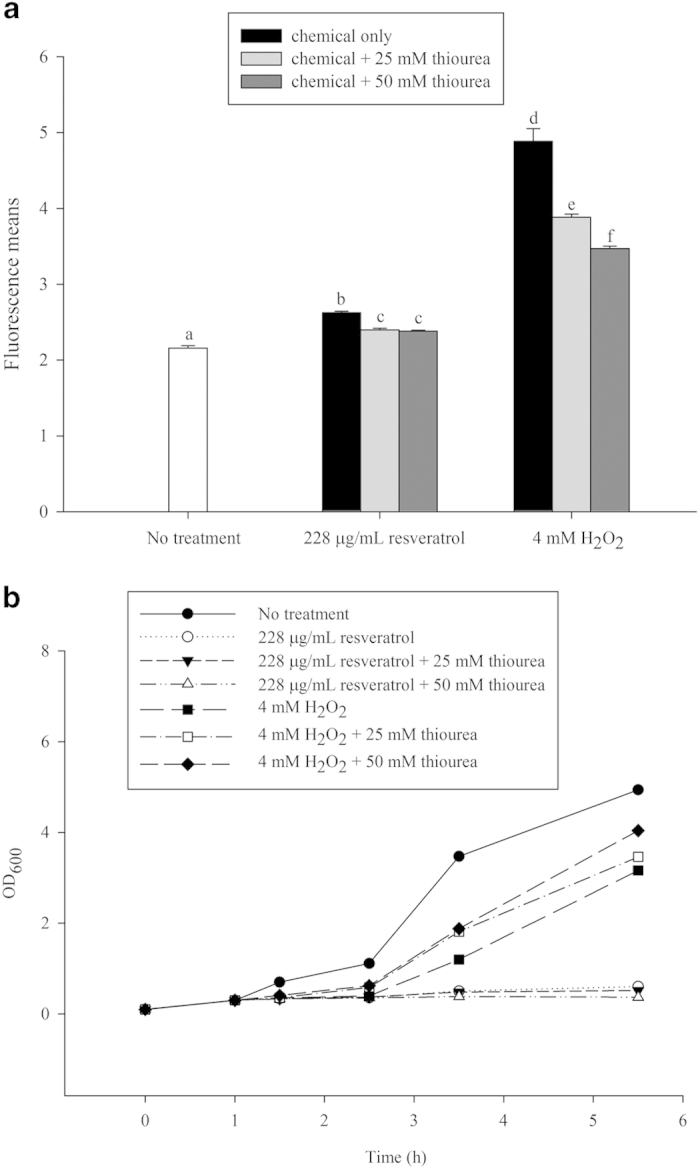
Cell growth recovery after addition of a ROS quencher (**a**) ROS production in cells treated with thiourea (ROS quencher). (**b**) ROS quencher-mediated recovery of *E. coli* growth. Differences among the groups were evaluated by analysis of variance (ANOVA) followed by Duncan’s multiple range tests. A level of *P* < 0.05 was considered statistically significant.

Fluorescence-activated cell sorting was performed to investigate ROS production in *E. coli* cells. H_2_O_2_ was used as a positive control. After resveratrol treatment, ROS significantly (*P* < 0.05) increased in a dose-dependent manner ( Figure S2). To investigate whether a direct correlation existed between ROS production and antibacterial activity, a ROS quencher, thiourea, was used. ROS production decreased significantly and dose-dependently in H_2_O_2_-treated cells with the addition of thiourea, a hydroxyl radical scavenger (Fig. 1a). In contrast, although ROS levels decreased significantly in resveratrol-treated cells after thiourea treatment, a dose-dependent decrease in ROS was not observed in these cells. In the H_2_O_2-_treated cells, cell growth was recovered by adding thiourea (Fig. 1b); however, growth of resveratrol-treated cells was not recovered by adding thiourea. This suggests that the increase in ROS following resveratrol treatment was not the major cause of cell death. Resveratrol-treated cell growth did not recover even when ROS levels decreased. In contrast, growth of H_2_O_2_-treated cells recovered when ROS levels decreased. These results indicate that resveratrol’s antibacterial activity is not related to ROS production.

### DNA fragmentation

**Figure 2:**
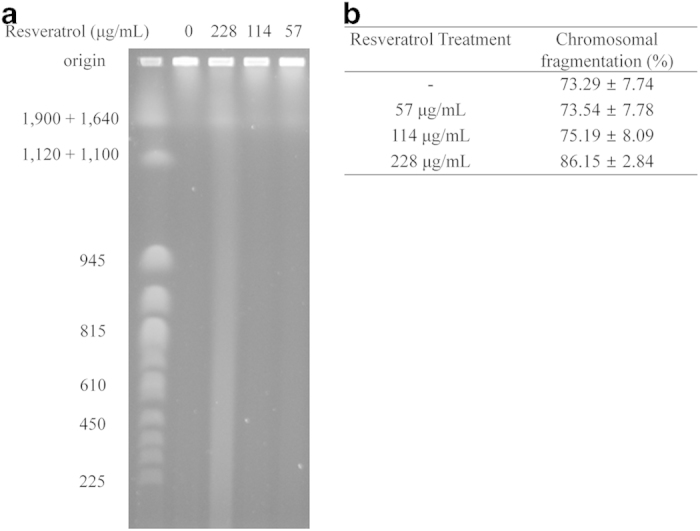
Effect of resveratrol on chromosomal DNA (**a**) Detection of chromosomal fragmentation by pulse-field gel electrophoresis. Bands in the chromosomal smears with an apparent molecular mass of 1,640–1,900 kbp correspond to the “compression zone” and are typical bands in a pulse-field gel of *E. coli* chromosomal DNA. Marker sizes (kbp) are shown on the left-hand side. DMSO treatment is the negative control. (**b**) Chromosomal fragmentation levels were quantified from pulse-field gels as the percentage ratio of the smear in the lane to the total signal (lane + well) from the sample using AlphaVIEW (Alpha Innotech Corporation, Version 3.0). Values are expressed as the mean ± SD (%) of three independent experiments.

To test whether resveratrol could induce DNA damage in *E. coli* cells, PFGE was conducted. Chromosomal DNA damage increased in resveratrol*-*treated *E. coli* cells in a dose-dependent manner (Fig. 2). DNA fragmentation increased 0.25%, 1.90% and 12.86 % in 0.125, 0.25 and 0.5 MIC resveratrol-treated cells, respectively, compared with the control. Cells treated with a sub-MIC (228 μg/mL) of resveratrol showed the most severe DNA damage. DNA fragmentation increased 1.2-fold in sub-MIC (228 μg/mL) resveratrol-treated cells compared with the control. This result suggests that resveratrol induced DNA fragmentation.

### SOS response to resveratrol

To investigate induction of the SOS response, we constructed an SOS response indicator strain containing the *lacZ*^+^gene under the control of the SOS-inducible promoter, *sulA*. To avoid gene dosage effects associated with multicopy vectors, *lac* fusions located on multicopy plasmids were converted into a single-copy fusion by homologous recombination. The resultant mutant, *E. coli* P90C-*sulA*, has only a single-copy chromosomal *sulA::lacZ* fusion expressing β-galactosidase activity proportional to the level of SOS induction. Resveratrol significantly (*P* < 0.05) increased SOS induction. The DNA crosslinking agent, mitomycin (0.5 μg/mL), was used as a positive control. The results showed that resveratrol (228 μg/mL) significantly (*P* < 0.05) induced SOS induction (Figure S3). SOS induction in sub-MIC (228 μg/mL) resveratrol-treated cells was higher than in mitomycin (0.5 μg/mL)-treated cells.

To confirm the effect of resveratrol on SOS induction, RT-PCR targeting 10 SOS response-related genes (*lexA*, *recA*, *uvrA*, *uvrB*, *uvrD*, *polB*, *dinB*, *umuD*, *soxR* and *sulA*) was performed. The results demonstrated that all SOS-related genes tested herein were overexpressed in a dose-dependent manner after resveratrol treatment (Figure S4). The expression levels of all the above genes increased 1.5-fold above those of the control when the cells were treated with a sub-MIC (228 μg/mL) of resveratrol.

### Morphological changes observed by phase-contrast microscopy

**Figure 3:**
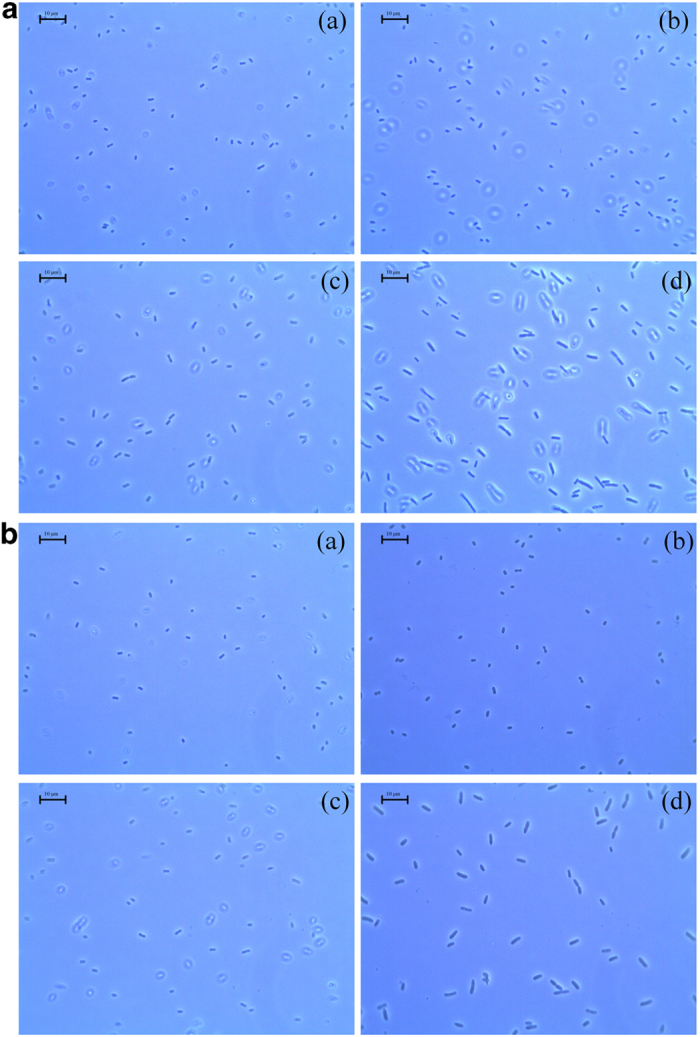
Phase-contrast microscopy Morphological changes in *E. coli* BW25113 (**a**) and *E. coli* JW0941 (**b**) were observed by phase-contrast microscopy. Cells treated with DMSO served as the negative control, no treatment (a), 57 μg/mL resveratrol (b), 114 μg/mL resveratrol (c) and 228 μg/mL resveratrol (d). Magnification, 1000 × .

Morphological changes in *E. coli* BW25113 were observed after resveratrol treatment. Untreated cells of appropriately 2 μm in size showed normal morphology (Fig. 3aa). Contrastingly, the lengths of the resveratrol-treated cells showed a dose-dependent increase in size (Fig. 3**a**b–d). Specifically, resveratrol treatment with a sub-MIC concentration (of 228 μg/mL) generated distinctly filamented cells of around 10–20 μm in length. Cell elongation increased about 5–10-fold compared with that of the control. This result suggests that resveratrol inhibited cell division and induced cell elongation by interfering with septum formation. Resveratrol treatment also induced cell elongation in a strain that lacks the SOS response in a dose-dependent manner (Fig. 3b). The results suggest that resveratrol inhibition of *E. coli* growth is not directly related to DNA fragmentation.

### Resveratrol inhibits Z-ring formation

**Figure 4:**
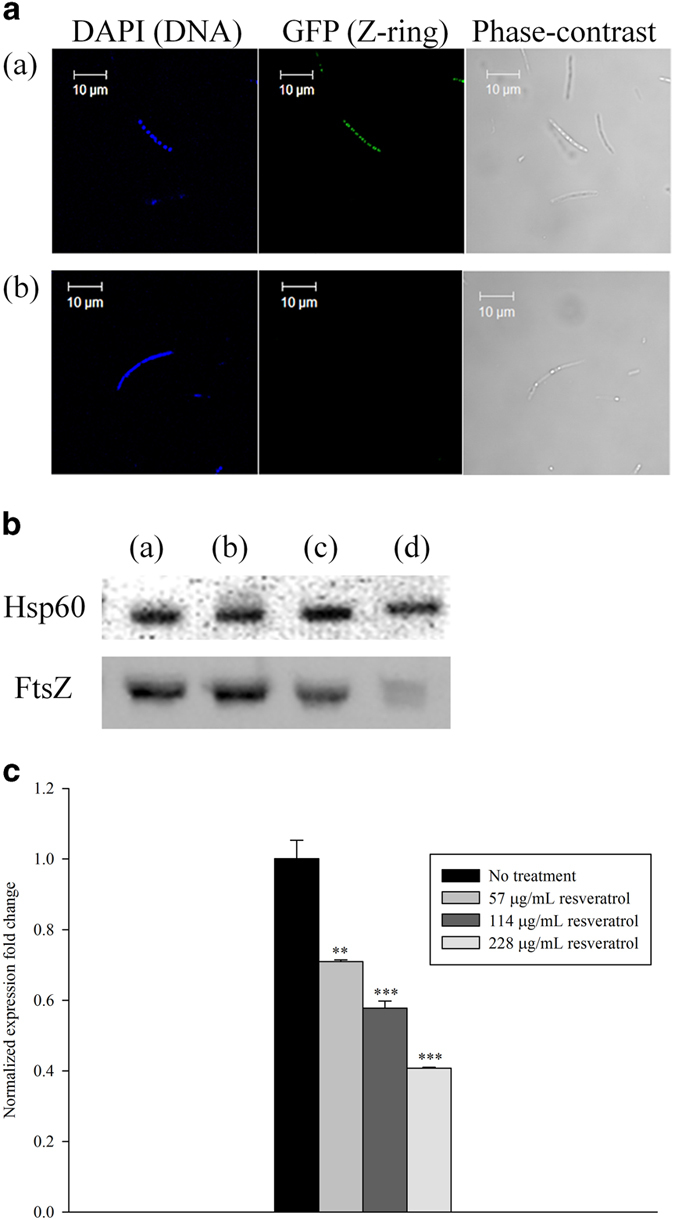
Detectionof Z-ring formation using confocal microscopy and western blot analysis of *E. coli*JW0093. (**a**) Cells were viewed by confocal microscopy at 1000 × magnification on an LSM 5 exciter confocal laser scanning microscope (Carl-Zeiss). (a), negative control treated with DMSO; (b), 228 μg/mL resveratrol treatment. (**b**) FtsZ expression levels measured by western-blot analysis. Hsp60 was used an endogenous control for *E. coli* and FtsZ was detected using an anti-His tag antibody. DMSO-treated cells were the negative control, no treatment (a), 57 μg/mL resveratrol (b), 114 μg/mL resveratrol (c) and 228 μg/mL resveratrol (d). (**c**) *ftsZ* expression measured by RT-PCR.

To confirm that the inhibitory effect of resveratrol was on Z-ring formation, we used an *E. coli* strain (JW0093) carrying pCA24N, a 6 × His-FtsZ-GFP fusion-containing plasmid. In the absence of resveratrol, most of the cells had one or more Z-rings (Fig 4aa). Without resveratrol treatment, *E. coli* clearly showed Z-ring formation. After resveratrol treatment at sub-MIC (228 μg/mL) most of the cells lacked Z-ring formation (Fig. 4ab). Therefore, to confirm the inhibition of FtsZ expression by resveratrol, western-blot analysis was performed. FtsZ levels decreased in the cells treated with resveratrol in a dose-dependent manner (Fig. 4b). The protein density of FtsZ in the control was set to 100%. The levels of FtsZ were approximately 93.8%, 81.2% and 61.7% after resveratrol treatment at 57, 114 and 228 μg/mL, respectively. The normalized ftsz expression fold-change decreased significantly (*P* < 0.05) in a dose-dependent manner (Fig. 4c). The expression of ftsz decreased 29.0%, 42.2% and 59.3% after resveratrol treatment at 57, 114 and 228 μg/mL, respectively. These results are consistent with the cell elongation results for resveratrol treatment and indicate that inhibition of Z-ring formation is a possible mode of action for resveratrol in *E. coli* cells.

### Resveratrol exhibits increased antibacterial activity in combination with PNA–*ftsZ*

**Figure 5:**
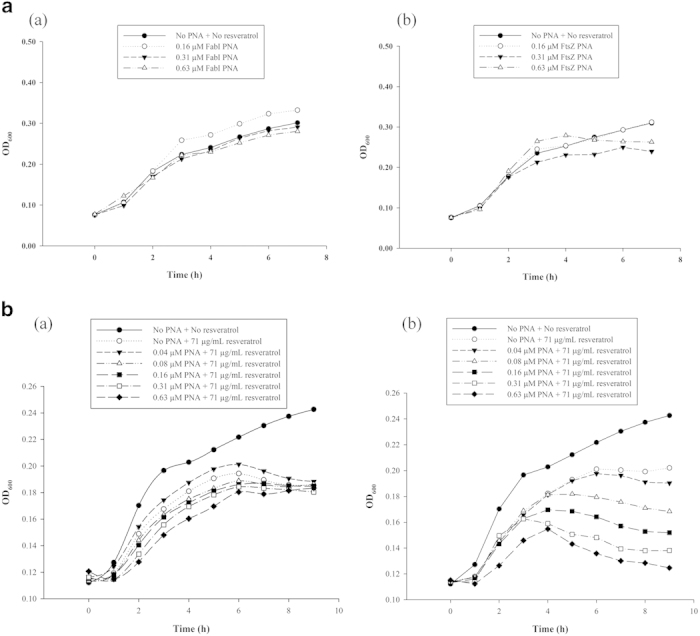
*ftsZ* silencing on cell growth of *E. coli*AS 19. *FabI*, a specific inhibitor of the enoyl-acyl carrier protein reductase, was used as a negative control (**a**) PNA alone treatment, (a) PNA- FabΙ and (b) PNA- FtsZ ; (**b**) PNA treatment with resveratrol (71 μg/mL), (a) PNA-FabI treatment and (b) PNA-FtsZ treatment with resveratrol.

**Figure 6:**
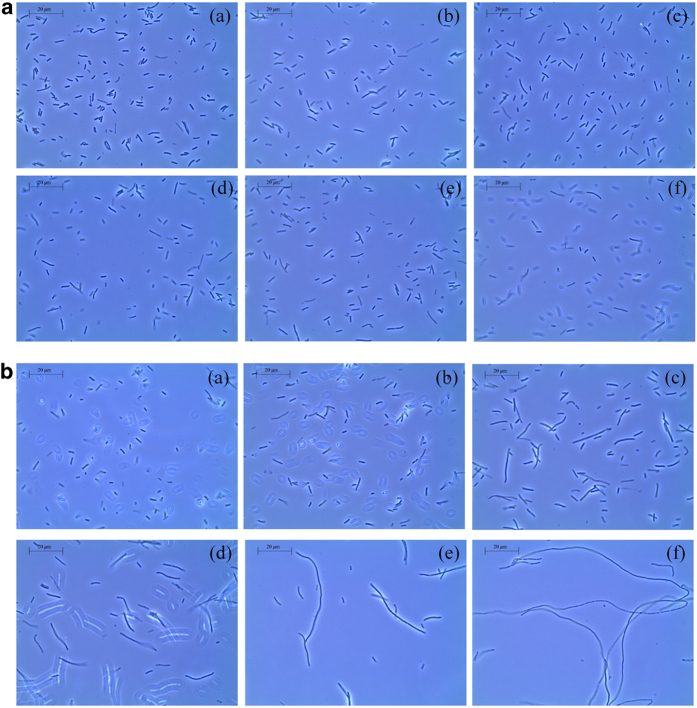
PNA-FtsZ treatment with resveratrol induces cell elongation (1000 × ). Morphological changes were observed by phase-contrast microscopy (**a**) PNA-FabI treatment. (**b**) PNA-*ftsZ* treatment. (a) negative control treated with 71 μg/mL of resveratrol alone; (b) resveratrol with 0.04 μM PNA; (c) resveratrol with 0.08 μM PNA; (d) resveratrol with 0.16 μM PNA; (e) resveratrol with 0.31 μM PNA; (f) resveratrol with 0.63 μM PNA.

To investigate whether PNA–*ftsZ* treatment makes *E. coli* more susceptible to resveratrol, we studied the synergetic effects between antisense PNA and resveratrol, because lowering (but not eliminating) expression of a target gene using antisense technology should cause the bacteria to be more susceptible to an antibacterial compound targeting the product of the gene^32^. The *fabI* gene encoding enoyl-ACP reductase, which catalyzes fatty acid elongation, was used as a negative control. We used the hyper-permeable *E. coli* strain AS19 to obtain efficient uptake of the different molecules. *E. coli* (AS19) growth was monitored every hour in the absence or presence of resveratrol or PNA. To confirm the synergic effect, the cells were treated with PNA alone (0.16, 0.31 and 0.63 μM). Silencing by treating with PNA alone showed no significant effect on cell growth (Fig. 5b). The MICs of resveratrol, PNA-*ftsZ* and PNA-*fabI* against *E. coli* AS19 were 142 μg/mL, 1.25 μM and 1.25 μM, respectively. We fixed the resveratrol concentration at 71 μg/mL (0.5 MIC). Addition of PNA–*ftsZ* suppressed bacterial growth in a dose dependent manner (Fig. 5bb). However, silencing *fabI* expression had no effect on cell susceptibility to resveratrol (Fig. 5ba). The results suggest that resveratrol inhibits cell growth by inhibiting FtsZ expression. Additionally, the effect of the gene silencers on cell morphology was investigated. PNA–fabI treatment did not affect cell morphology (Fig. 6a), while treatment with PNA–*ftsZ* promoted cell elongation in a dose dependent manner (Fig. 6b). These results suggest that resveratrol inhibits cell growth by inhibiting FtsZ expression. The results from *ftsZ* silencing and cell elongation suggest that resveratrol directly targets the bacterial division protein FtsZ.

## Discussion

Antimicrobial activities of antioxidants from natural sources have been investigated and many of them have not revealed their mechanism(s) action. Many studies have investigated the relationship between ROS and antibacterial activity in bacteria^33^. Currently, controversy exists about whether ROS formation contributes to cell death in *E. coli*. Kohanski *et al.*^34^ reported that, regardless of drug-target interactions, antibiotics stimulate the production of highly deleterious hydroxyl radicals in Gram-positive and Gram-negative bacteria, which ultimately induces cell death. This theory was widely accepted and became established wisdom. However, some recent evidence refutes a role for ROS in bacterial pathogen killing^35^,^36^. These studies reported that no direct correlation between ROS and antibacterial activity exists. In the present study, however, resveratrol showed antibacterial activity against *E. coli* and induced ROS production in this bacterium. The ROS level in resveratrol-treated cells increased, as did the antibacterial activity. However, ROS quenching experiments revealed that the antibacterial activity of resveratrol was not directly related to ROS production. ROS induce oxidative damage to DNA such as strand breaks and nucleotide modifications, particularly in sequences with a high guanosine content^37^. In *E. coli*, DNA damage induces the expression of more than 40 genes leading to the arrest of cell division, DNA repair, prophages, toxin production and mutagenesis^38^. Increasing ROS levels, in turn, induce the SOS response, an inducible DNA repair system allowing bacteria to survive following sudden increases in DNA damage. There are some reports that several classes of plant-derived polyphenolic compounds such as flavonoids^39^, tannins^40^, curcumin^41^ and capsaicins^42^ are capable of causing oxidative DNA damage. ROS, especially hydroxyl radicals, are extremely toxic and will readily damage protein, membrane lipids, and DNA^43^. In the present study, resveratrol clearly induced DNA fragmentation at a concentration of 228 μg/mL, which might be directly or indirectly related to its ability to enhance ROS production. Although the mechanism has not been clearly shown, our results suggest that resveratrol might act in itself as a DNA-cleavage agent.

DNA damage activates the coprotease activity of the RecA protein; this promotes self-cleavage of the SOS transcriptional repressor LexA, and leads to increased transcription of the SOS response regulon^44^,^45^. Here, we found that resveratrol treatment significantly (*P* < 0.05) increased the SOS response, which is the major system for mending chromosomal lesions in *E. coli*. Resveratrol treatment of *E. coli* induced over-expression of SOS response-related genes (i.e., *lexA*, *recA*, *uvrA*, *uvrB*, *uvrD*, *polB*, *dinB*, *umuD*, *soxR* and *sulA*). The results show that resveratrol-induced DNA fragmentation caused induction of the SOS response to cope with DNA fragmentation. This finding might explain why resveratrol has an antibacterial effect. Bacterial filamentation has often been observed as a result of cellular responses to various stressors, including DNA damage and inhibition of DNA replication. Resveratrol treatment promoted cell elongation in *E. coli* in a dose-dependent manner. However, unexpectedly, an SOS response-negative strain also showed cell elongation following resveratrol treatment. These results imply that the antibacterial activity of resveratrol may occur via a process leading to DNA fragmentation. Indeed, *E. coli* treated with resveratrol became elongated with or without an SOS response.

Filamentation is an abnormal growth characteristic in *E. coli* whereby cells continue to elongate but do not divide because no septa has been formed. Cell elongation is caused by inhibition of cell division via a block in Z-ring formation, a process essential to cell division. FtsZ is a key protein for septum formation when cells are dividing. The SOS-related gene, *sulA*, is upregulated by DNA damage and its product binds to FtsZ to prevent septum formation resulting in cell division inhibition until the damaged DNA is repaired^46^; following this, SulA is proteolyzed^31^. We found that cells treated with resveratrol experienced severely interrupted Z-ring formation, suggesting that FtsZ might be the main target for resveratrol in *E. coli* via suppression of *ftsZ* expression. Two antisense inhibitors, PNA-*ftsZ* and PNA-*fabI*, were used to test this conjecture. PNA is an artificially synthesized polymer similar to DNA or RNA; however, its high stability makes it a powerful research tool for conditional and titratable reduction of expression of specific genes^47^. Here, PNA-*ftsZ* treatment combined with resveratrol had a synergistic effect on antibacterial activity, while PNA-*fabI*, an antisense inhibitor of an essential gene, did not show this synergistic effect. This indicates that resveratrol inhibits cell growth by suppression of *ftsZ* expression. Berberin, an alkaloid produced by several plant species, was shown to have antioxidant properties^48^, and exhibit antibacterial activity through inhibition of the cell division protein FtsZ^32^. A possible mechanism of action for resveratrol in bacteria has been reported: in a study on two species of *Arcobacter*, resveratrol inhibited DNA synthesis leading to an increase in cells with lower DNA content and impaired cell division^16^. Taken together, our findings provide the first indication that resveratrol’s mechanism of action against *E. coli* is mediated by FtsZ targeting.

In conclusion, resveratrol treatment led to DNA fragmentation in *E. coli*, which induced an SOS response; however, resveratrol also induced cell elongation without an SOS response. Resveratrol inhibited Z-ring formation in *E. coli* cells. Our results suggest that regardless of SOS response induction, resveratrol suppresses FtsZ expression and Z-ring formation eventually leading to inhibition of cell growth. FtsZ protein plays an essential role in Z-ring formation in *E. coli* and has a high level of species conservation in bacteria. Therefore, FtsZ is a promising target for antibacterial drug development.

## Methods

### Quantification of chromosomal fragmentation using pulse-field gel electrophoresis (PFGE)

An overnight *E. coli* (BW25113) culture was diluted to an OD_600_ of 0.1 and incubated for 1 h at 37 °C with shaking at 200 rpm. Resveratrol (57, 114 and 228 μg/mL) and DMSO (control) were added to 20 mL of diluted culture followed by incubation at 37 °C for 4 h with shaking at 200 rpm. Cell cultures treated in this manner were harvested by centrifugation at 5,000 × *g* for 10 min at room temperature and washed with 50 mM EDTA (twice) followed by resuspension in EC buffer (6 mM Tris, 1 M sodium chloride, 100 mM EDTA, 0.5% w/v Brij58, 0.2% w/v sodium deoxycholate and 0.5% v/v sarkosyl, pH 7.5). At this stage the cell density of the individual samples was adjusted to obtain an equal cell density per sample (using spectrophotometer at 600 nm). Cell suspensions (100 μL) from these samples were transferred to sterile 1.5 mL micro-centrifuge tubes. Next, 100 μL of molten 1.2% chromosomal grade agarose was added followed by brief mixing, after which the solution was dispensed immediately into a plug mold, then left in the sample block for 10 min. The block was transferred to a 1.5 mL microcentrifuge tube containing 500 μL of EC buffer and 50 μL of lysozyme (10 mg/mL in 10 mM Tris-HCl, pH 8.0) and the tube was incubated at 37 °C for 4 h. After incubation, EC buffer was removed and replaced with 300 μL of EST buffer (5 mM Tris, 0.5 M EDTA and 1% v/v sarkosyl, pH 7.5) and 30 μL of proteinase K (20 mg/mL), after which the block was incubated at 50 °C overnight. The blocks were washed with 1 mL of 50 mM EDTA (three times) and were then loaded into wells made with the 1% Certified Megabase agarose gel (Bio-Rad Laboratories, Hercules, CA, USA) in 0.5 × TBE (25 mM Tris, 25 mM boric acid and 20 mM EDTA). Electrophoresis was performed at 14 °C at 200 V (6 V per cm) for 22 h with pulsing times of 50–80 sec using a CHEF-DR II pulse field gel electrophoresis instrument (Bio-Rad). On completion of the electrophoresis, the gel was stained with ethidium bromide solution (0.5 μg/mL) and destained in sterile deionized water for 1 h^49^.

### Morphology change observations

Phase-contrast microscopy was performed to check for morphological changes in the cells after exposure to resveratrol. The *E. coli* strains grown overnight were diluted to an OD_600_ of 0.1 and were then incubated at 37 °C for 1 h with shaking at 200 rpm. Resveratrol (57, 114 and 228 μg/mL) and DMSO (control) were added to the 20 mL of diluted culture and incubated at 37 °C with shaking at 200 rpm. After 4 h, the cells were collected by centrifugation at 5,000 × *g* for 5 min. Next, the pellets were washed twice with PBS and 10 μL samples were placed onto glass slides. Microscopic images were acquired by Leica DM750 phase-contrast microscopy (Leica, Wetzlar, Germany).

### Detection of inhibition of Z-ring formation using confocal microscopy

To investigate Z-ring formation in *E. coli*, we used *E. coli* JW0093. An overnight culture of *E. coli* JW0093 was diluted to an OD_600_ of 0.1 and incubated at 37 °C with shaking at 200 rpm. When the cells reached exponential phase, resveratrol was added to 20 mL of diluted culture. After 1 h, to induce green fluorescent protein (GFP) controlled by the *lac* promoter, 1 mM isopropyl β-D-thiogalactopyranoside was added. The cells were collected by centrifugation after 4 h incubation and washed three times with an equal volume of PBS to remove residual growth medium and chemicals, and the cells were resuspended in 1 mL of PBS. 4′,6-diamidino-2-phenylindole (DAPI, Sigma) was added to a final concentration of 1 μM and the cells were incubated for 10 min at room temperature. Excess dye was removed by three washes with PBS, a 10 μL sample of the cells was placed onto a slide and a cover slip was mounted onto it using VECTASHIELD (Vector Laboratories, Burlingame, CA, USA). The cells were observed by confocal microscopy at 1,000 × magnification on a LSM 5 exciter confocal laser scanning microscope (Carl-Zeiss, Jena, Germany).

### Western blot quantification of FtsZ protein levels

To measure FtsZ protein levels, western blot analysis was performed. The N-terminal of the FtsZ-GFP fusion protein was tagged with 6 × His. Sample preparation was performed by the same procedure used for confocal microscopy. The cells collected were treated B-PER bacterial protein extraction reagent (Thermo Scientific) for 10–15 min at room temperature and then centrifuged at 15,000 × *g* for 5 min to separate the soluble and insoluble proteins. Soluble proteins (30 μg per lane) were separated by 12.5% SDS-polyacrylamide gel electrophoresis. The separated proteins were transferred to a polyvinylidene difluoride membrane. The membrane was blocked with 5% non-fat skimmed milk in PBS with 0.05% Tween 20 (PBS-T) overnight at 4 °C. The membrane was washed three times with PBS-T and then incubated with a 1:5,000 dilution of an anti-His antibody (Invitrogen) for 1 h at room temperature. Heat shock protein 60 (Hsp60), used as an endogenous control for *E. coli*, was detected with a 1:1,000 dilution of anti-Hsp60 antibody (Thermo Scientific). The membrane was washed three times with PBS-T, and incubated further with a secondary antibody (horseradish peroxidase-conjugated anti-mouse IgG antibody, AbFrontier, Seoul, Korea) at a 1:10,000 dilution for 1 h at room temperature. Bound antibodies were detected with an enhanced chemiluminescence (ECL) reagent kit (Perkin Elmer, Boston, MA USA). Protein density was quantified using AlphaView SA software (Cell Biosciences, Santa Clara, CA, USA).

### *ftsZ* RNA silencing using *ftsZ*-specific antisense peptide nucleic acid (PNA)

The *E. coli* strain AS19 (kindly provided by Professor Peter E. Nielsen) with a hyper-permeable phenotype has been used for RNA silencing^47^. The structures of the PNA molecules are shown in Supplemental Table 3 (manufactured by Panagene, Korea). To enhance cell uptake, the carrier peptide (KFF)_3_K was attached to each PNA molecule^50^,^51^. To investigate the PNA–FtsZ and resveratrol combination, the MICs of the PNAs were determined against *E. coli* AS19 using the broth microdilution method described above. For the RNA silencing assay, PNA was dissolved in distilled water and 10 μL of the diluted PNA (from 0.53 μM to 0.04 μM) was dispensed into each well of a 96-well plate, 180 μL of cells (OD_600_ is 0.1) were added, and the plate cultured in Muller–Hinton broth (Difco, Detroit, MI, USA) overnight followed by the addition of 10 μL of resveratrol at a designated concentration. Bacteria were cultured at 37 °C in a SPECTRA max microplate reader (Molecular Devices) and cell growth was monitored by measuring the OD_600_ every 1 h.

### Statistical analyses

All statistical analyses were performed using SPSS version 12.0 for Windows (SPSS, Chicago, IL, USA). Values are expressed as the mean ± SD (standard deviation) of three independent experiments performed in triplicate. The statistical significance of difference was determined with the Student’s *t* test. A *P*-value of < 0.05 was considered the threshold for statistical significance. Statistical differences among groups were evaluated by analysis of variance (ANOVA), followed by Duncan’s multiple range tests. A level of *P* < 0.05 was considered statistically significant.

## Author Contributions

Conceived and designed the experiments: D.H. and Y.H.L. Performed the experiments: D.H. and Y.H.L. Analyzed the data: D.H. and Y.H.L. Contributed reagents/materials/analysis tools: D.H. and Y.H.L. Wrote the paper: Y.H.L.

## Additional Information

**How to cite this article**: Hwang, D. and Lim, Y.-H. Resveratrol antibacterial activity against Escherichia coli is mediated by Z-ring formation inhibition via suppression of FtsZ expression. *Sci. Rep.* **5**, 10029; doi: 10.1038/srep10029 (2015).

## Supplementary Material

Supplementary Information
