# Supplementary material for: Resveratrol antibacterial activity against Escherichia coli is mediated by Z-ring formation inhibition via suppression of FtsZ expression
Source: Sci Rep. 2015 May 5;5:10029. doi: 10.1038/srep10029 (PMC4419592; doi:10.1038/srep10029)
Supplement: Supplementary Information [file srep10029-s1.pdf]

Supplemental information

Resveratrol antibacterial activity against *Escherichia coli* is mediated by Z-ring formation inhibition via suppression of FtsZ expression

Dahyun Hwang[1] and Young-Hee Lim[1,2*]

1. Department of Public Health Science (BK21 PLUS program), Graduate School, Korea University,
2. Department of Laboratory Medicine, Korea University Guro Hospital, Seoul, South Korea

*Corresponding author

E-mail: yhlim@korea.ac.kr

Experimental Section

Bacteria strains and culture conditions. The origins of the *Escherichia coli* strains, DNA constructs and phage used in this work are described in Supplemental Table 1. *E. coli* were grown in Luria–Bertani (LB) broth (Difco, Sparks, MD, USA) or on LB agar plates. For phage growth, bacteria were grown in λym medium (10 g tryptone, 1 g yeast extract, 2.5 g NaCl, and 2 g maltose per liter, pH 7.2) or on a λ plate (10 g tryptone, 2.5 g NaCl and 10 g agar per liter) [1]. Antibiotics and other supplements were used in the following concentrations: 100 μg/mL ampicillin; 30 μg/mL chloramphenicol; X-gal 0.04 mg/mL in plates and 0.4 mg/mL in λ top agar.

Minimal inhibitory concentration (MIC) tests. The MIC of resveratrol against *E. coli* BW25113 was measured using the microbroth dilution method (CLSI, 2008) of the Clinical and Laboratory Standards Institute (CLSI; formerly NCCLS). Resveratrol (Sigma, St Louis, MO, USA) was dissolved in dimethyl sulfoxide (DMSO), and after making serial double dilutions, the resveratrol concentration ranged from 0.4−456 μg/mL. Each experiment included a positive control (DMSO only) and a negative control (no bacterial cells). Freshly cultured *E. coli* BW25113 was added to each dilution at a final concentration of 5×10^5 colony forming units-(cfu)/mL of cells. After incubating the *E. coli* cells at 37℃ for 24 h with shaking at 200 rpm, the MIC was determined. For the growth inhibition assay, *E. coli* BW25113 was inoculated into LB broth and incubated overnight at 37℃ with shaking at 200 rpm. The overnight culture was diluted to an optical density (OD_{600}) of 0.1 and resveratrol was added at a concentration equivalent to 0, 0.125, 0.25, 0.5 and 1 MIC (μg/mL) per 20 mL of diluted culture, after which the cultures were incubated at 37℃ for 12 h with shaking at 200 rpm. Viable cells were counted every 2 h by 1 mL sampling followed by 10-fold dilution

with saline and plating on LB agar. Plates inoculated in this manner were incubated overnight and the numbers of colonies on each plate were counted.

ROS measurements. Endogenous amounts of ROS were measured by a fluorometric assay with dichlorofuorescein diacetate (H_2DCF-DA) (D-399, Invitrogen, Carlsbad, CA, USA), according to the method of Kobayashi with a minor modification [2]. Briefly, an overnight culture of *E. coli* BW25113 was diluted to an OD_{600} of 0.1 and then incubated for 1 h at 37℃ with shaking at 200 rpm followed by addition of 114 μg/mL to 228 μg/mL of resveratrol or 4 mM of H_2O_2 (positive control) to the 20 mL of diluted culture. For the hydroxyl radical quenching experiments, 25 mM or 50 mM thiourea was added simultaneously to each culture followed by incubation at 37℃ for 4 h with shaking at 200 rpm. The 1 mL cultures (adjusted to an OD_{600} of 1) were each transferred to 1.5 mL micro-centrifuge tubes and the cells were harvested by centrifugation at 5,000 × *g* for 10 min at room temperature. Then, cells were washed with phosphate buffered saline (PBS) three times, and 10 μL of H_2DCF-DA (2.5 mM) was added. The samples were incubated at room temperature for 30 min with gentle shaking. After incubation, the cells were harvested by centrifugation at 5,000 × *g* for 10 min at 4℃ and washed with 500 μL of PBS (twice) to remove the excess dye. ROS production from 50,000 cells per sample was measured by BD FACSCalibur flow cytometry (BD Biosciences, San Jose, CA, USA) (excitation: 488 nm, emission: 525 nm).

SOS induction measurements. To investigate induction of the SOS response in *E. coli*, we assembled a single-copy chromosomal *sulA::lacZ* fusion construct. The genomic DNA of *E. coli* BW25113 was used as a template and the *sulA* promoter was PCR amplified using the following primers: forward primer containing an *Eco*RI site 5′-CG GAA TTC GAT TTC GCC ATA GAC TTT C and reverse primer containing a *Bgl*II site 5′-GA AGA TCT GAT AAG CCC GGC TGT AGT G. The PCR amplification conditions comprised denaturation at

95°C for 10 min followed by 30 cycles of 20 sec at 95°C, 30 sec at 58°C and 30 sec at 72°C and a final extension at 72°C for 5 min. The resulting PCR fragments were digested with *Eco*RI and *Bgl*II and directionally cloned into the *Eco*RI–*Bam*HI digested *lacZ* operon fusion vector, pRS415. The amplified *sulA* fragment contains a *Bam*HI restriction site; hence, the reverse primer was flanked by a *Bgl*II site (Figure S5) [3]. After ligation, the plasmid was transformed into the *lac*-defective host strain, *E. coli* P90C. The selected colony was checked by nucleotide sequencing. The pRS415 plasmid-borne *sulA::lacZ* fusion was transferred to a non SOS-inducible λRS88 *ind*$^{-}$ λ phage vector by homologous recombination, *in vivo*. This phage contains sequences homologous to *lacA*, *lacY*, and part of the *lacZ* gene of pRS415, thereby allowing *in vivo* recombination to occur between the plasmid and phage. The insert-containing progency phage was then used to infect P90C to produce lysogens via integration into the *sulA* gene promoter resulting in formation of a fuctional *sulA::lacZ* operon fusion from which the subsequent lysogens were selected on LB + X-gal plates [1]. SOS induction levels were measured by assaying the activity of β-galactosidase from *E. coli* P90C-sulA [4]. An overnight culture of this strain was diluted to an OD_{600} of 0.1 and incubated at 37°C for 1 h with shaking at 200 rpm. Resveratrol (114 μg/mL and 228 μg/mL) or 0.5 μg/mL of the positive control (mitomycin) were added to the samples and they were incubated at 37°C for 1 h with shaking at 200 rpm. The cells were harvested by centrifugation at 4°C for 10 min at 5,000 × *g*. Samples (500 μL) were diluted to an OD_{600} of 0.5 and then washed twice with PBS. To determine the culture density, each sample (180 μL) was transferred to 96-well microplate and the OD_{600} measured using a SPECTRA max microplate reader (Molecular Devices, Sunnyvale, CA, USA). To facilitate cell permeabilization, 320 μL of buffer Z (60 mM $Na_2HPO_4 \cdot 7H_2O$, 40 mM $NaH_2PO_4 \cdot H_2O$, 10 mM KCl, 1 mM $MgSO_4 \cdot 7H_2O$ and 50 mM β-mercaptoethanol) was mixed with the remaining 320 μL of the cells, and 20 μL of freshly

prepared 0.1% SDS and 40 μL of chloroform were added, followed by a 10 sec vortex. After allowing the chloroform to settle to the bottom of the tube, 180 μL of the permeabilized cells were transferred to a 96-well microplate containing buffer Z. The assay was initiated by adding 20 μL of *O*-nitrophenyl-β-D-galactoside (ONPG) (4 mg/mL) to each well of the plate [5]. Then, the OD_{420} and OD_{550} of the samples were measured for 5 min. The β-galactosidase activity was calculated as follows: $1000 \times (OD_{420} - 1.75OD_{550})/(t \times v \times OD_{600})$, where t is the reaction time (in min), and v is the volume of culture used in the assay (in mL).

Quantitative real-time reverse transcription-polymerase chain reaction (RT-PCR). An overnight culture of *E. coli* BW25113 was diluted to an OD_{600} of 0.1 and then incubated for 1 h at 37℃ with shaking at 200 rpm. Resveratrol (114 and 228 μg/mL) was added to 20 mL of diluted culture and the cells were incubated at 37℃ with shaking at 200 rpm. After 1 h, the cells were collected by centrifugation at 5,000 × *g* for 10 min. Total cellular RNA was prepared with a Pure Link RNA mini kit (Invitrogen), according to the manufacturer's protocol. The RNA concentration was quantified with a NanoDrop ND-1000 Spectrophotometer (Thermo Scientific, Wilmington, DE, USA). Next, cDNA was prepared using a Revert Aid First Strand cDNA kit (Fermentas, Germany), and quantitative PCR (qPCR) was performed with a DyNamo™HS SYBR Green qPCR kit (FINNZYMES, Finland) using a StepOnePlus™ Real-Time PCR System (Applied Biosystems, Foster City, CA, USA). The sequences of the primer sets used for real-time PCR are shown in Supplemental Table 2. The reaction was preheated at 95℃ for 10 min followed by 40 cycles at 95℃ for 20 sec, 56℃ for 20 sec and 72℃ for 30 sec. The reaction without cDNA served as a negative control. The real-time PCR data was quantified based on the number of cycles needed for amplification-generated fluorescence to reach a specific detection threshold (the Ct value). Relative gene expression was quantified on the basis of equal amounts of RNA (1 μg) and the

average Ct value for each gene, where delta Ct ($\Delta Ct = Ct_{target\ gene} - Ct_{reference\ gene}$) was calculated from the Ct values of genes in the same sample. 16s rRNA was used as the internal control reference gene. The ΔΔCt value was calculated with the equation $\Delta\Delta Ct = (\Delta Ct_{treated} - \Delta Ct_{untreated})$. The normalized expression change was expressed as $2^{-\Delta\Delta Ct}$ (the 16s rRNA control was set to 1) [6].

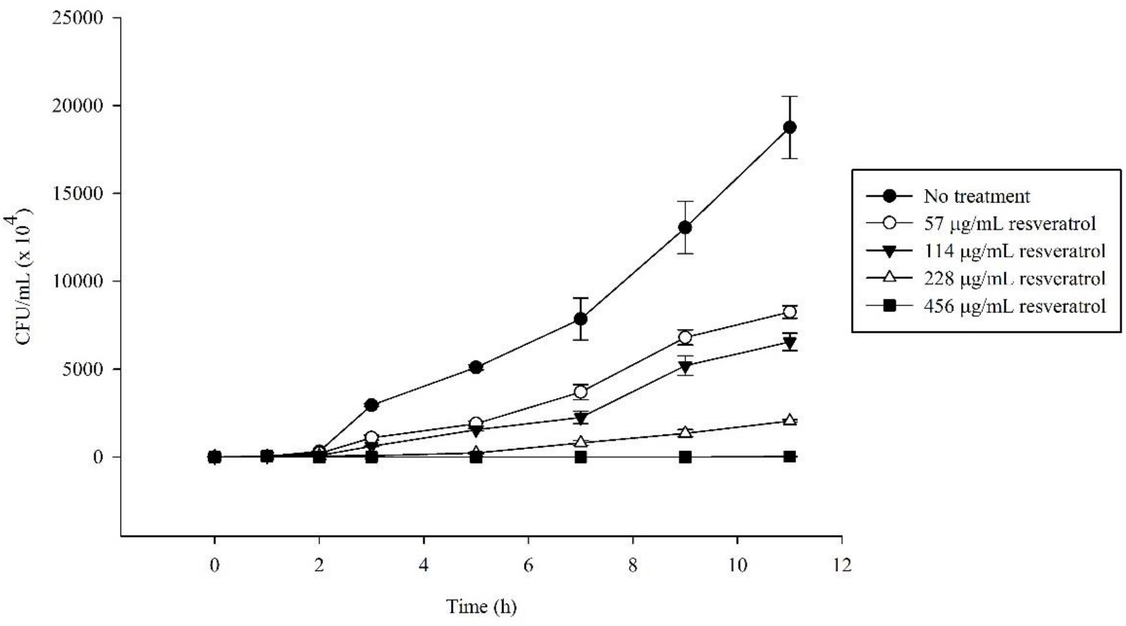

Figure S1. Growth inhibition by resveratrol.

Values are expressed as the mean ± SD of three independent experiments.

CFU, colony forming units.

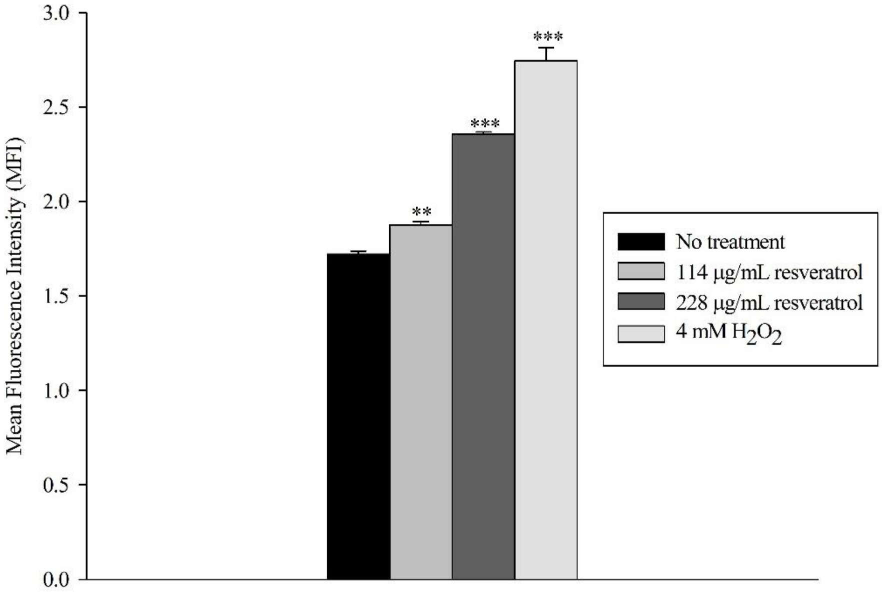

Figure S2. Flow cytometric analysis of ROS levels. Fluorescence denotes 50,000 bacterial cells treated with resveratrol at 114 µg/mL and 228 µg/mL, 4 mM H_2O_2 (positive control) or the negative control (denoted here as no treatment). Mean fluorescence intensity of 50,000 bacterial cells was calculated for each sample and values are expressed as the mean ± SD of three independent experiments. $^{**}P < 0.01$, $^{***}P < 0.001$, Student's *t*-test, compared with control.

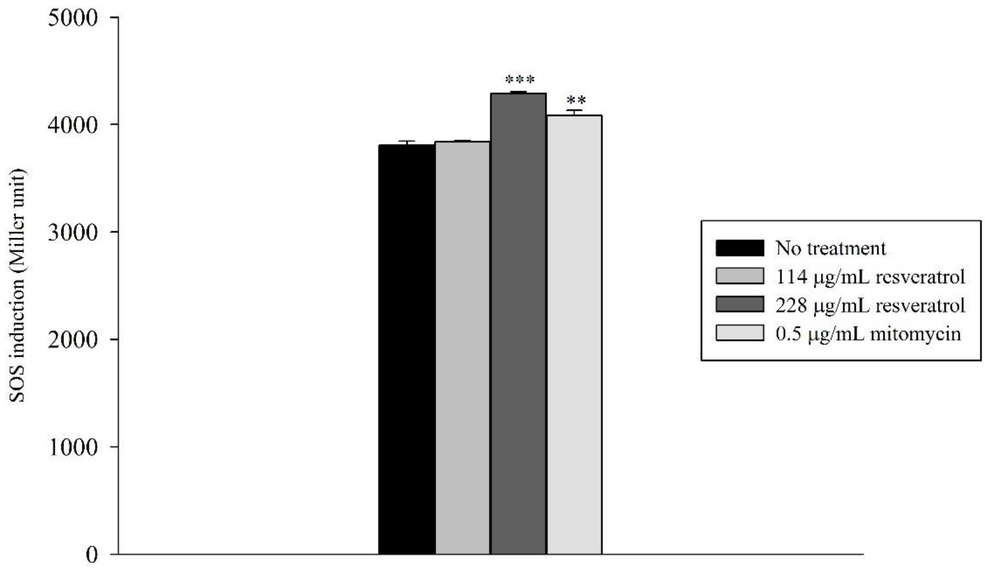

Figure S3. SOS induction. Activation of the SOS responses was monitored using the engineered construct, *E. coli* P90C-*sulA*. Total β–galactosidase activity was determined as described by Miller[4]. Values are expressed as the mean ± SD of three independent experiments. **P < 0.01, ***P < 0.001, Student's *t*-test, compared with control.

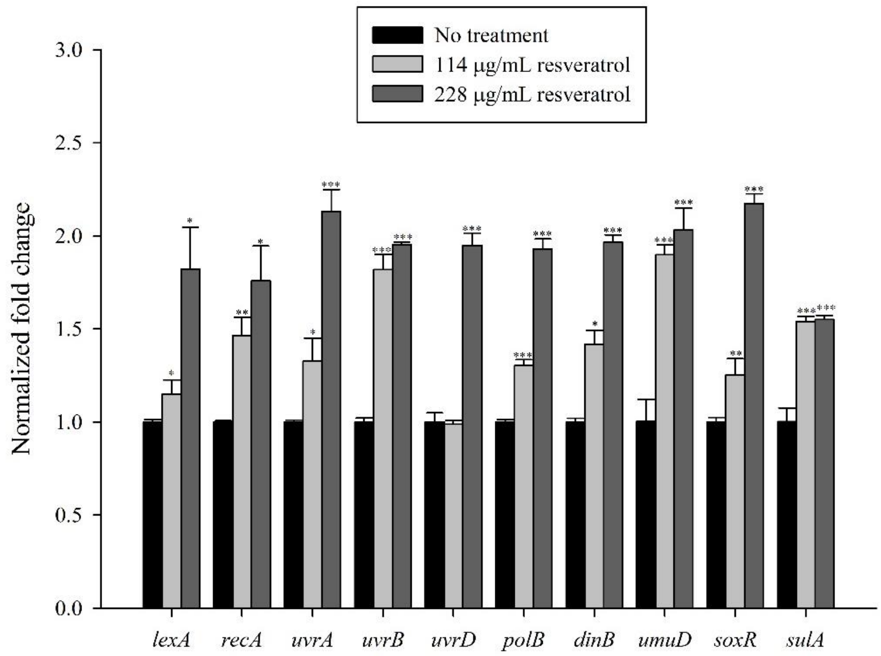

Figure S4. Effect of resveratrol on SOS-related gene expression. The real-time PCR data was quantified based on the number of cycles needed for amplification-generated fluorescence to reach a specific threshold of detection (the Ct value). Values are expressed as the mean ± SD of three independent experiments performed in triplicate. **P* < 0.05, ***P* < 0.01, ****P* < 0.001, Student's *t*-test, compared with control.

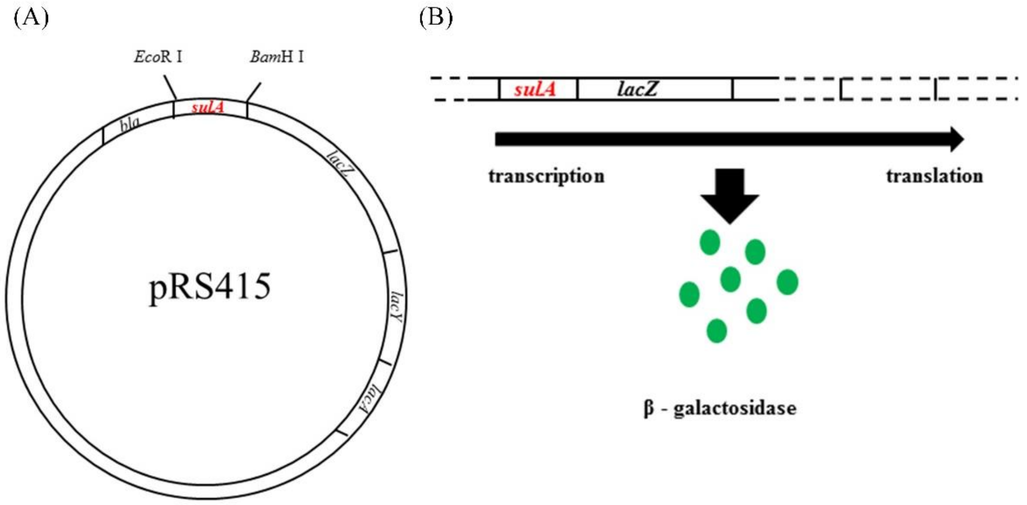

Figure S5. Construction of the single-copy promoter-*lacZ* fusion-containing strain, pRS415.

(A) pRS415 was constructed to facilitate expression of fusion proteins from the *E. coli lacZ* gene. pRS415 has a promoterless *lacZYA* operon for creating gene fusions and can be converted to single-copy fusions by recombination with lambda phage. pRS415 was digested with *Eco*RI and *Bam*HI and amplified *sulA* fragments were digested with *Eco*RI and *Bgl*II and then ligated to *Bam*HI and *Bgl*II (compatible sites) in the plasmid. (B) The phage contained sequences homologous to *lacA*, *lacY* and part of the *lacZ* gene of pRS415, thus allowing *in vivo* recombination to occur between the plasmid and phage. The insert-containing progeny phage was used to infect P90C for lysogen production. The complete *sulA::lacZ* operon fusion lysogene has just one copy (chromosomal) of this reporter system. We called the completed construct *E. coli* P90C-*sulA*. The β-galactosidase activity produced by this construct is proportional to expression of the *sulA* promoter.

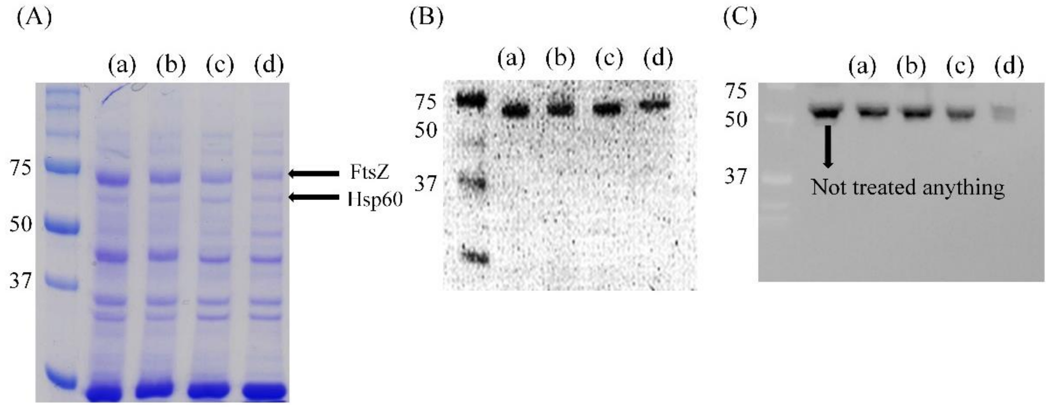

Figure S6. Full scans of panel from Figure 4.

(A) Coomassie blue staining of SDS-PAGE gel. (B) Western blot for Hsp60. (C) Western blot for FtsZ. DMSO-treated cells were the negative control, no treatment (a), 57 µg/mL resveratrol (b), 114 µg/mL resveratrol (c) and 228 µg/mL resveratrol (d).

Supplementary Table 1 *Escherichia coli* strains, plasmids and phage used in this work

Strain, phage or plasmid	Relevant characteristic(s) or genotype	Reference
E. coli BW25113	K-12, BD792 derivative	[7]
E. coli P90C	F-, *ara-600*, *Δ(gpt-lac)5*, λ^-, *relA1*, *spoT1*, *thi-1*	[8]
E. coli AS19	LSP defect	[9]
E. coli JW0941	*ΔsulA773::kan*, *rph-1*, *Δ(rhaD-rhaB)568*, *hsdR514*	[10]
E. coli JW0093(ASKA+)	ColEI, PT5-lac::6×His-FtsZ-GFP *cat*	[11]
pRS415	*lacZ*$^+$ *lacY*$^+$ *bla*$^+$	[1]
λRS88	λ *imm434 ind*$^-$ *bla'* -*lacZ*$^+$ *lacY*$^+$	[1]
E. coli P90C-*sulA*	*sulA::lacZ*	This study

Supplementary Table 2 Real-time PCR primers

Gene names	Gene ID	Product size (bp)	Forward primer	Reverse primer
16s rRNA	944939	204	CAGCCACACTGGAACTGAGA	GTTAGCCGGTGCTTCTTCTG
lexA	948544	163	CTTGCTGGCAGTGCATAAAA	CTGACGAAGGTCAACGACAA
recA	947170	158	GGCCGTATCGTCGAAATCTA	ATATCGACGCCCAGTTTACG
uvrA	948559	183	TTTCAGGTTCCGGTAAATCG	CCGGGTTAGAACGTGGAGTA
uvrB	945385	177	AGCGATTCTCTACGGCGATA	GCTTTGGTTTTGGCAATGTT
uvrD	948347	213	ACCAATATCCTGGTGGACGA	AGAGCGGTAGTTTTGCTCCA
polB	944779	216	GCCGATGAAGAAAACCAAAA	AAGTTGCCCGGTCATAAGTG
dinB	944922	176	ACGCCTACAAAGAAGCCTCA	TTGCAGCTCGTTGAAGATTG
umuD	945746	248	CTCCGCGAAATTGTGACTTT	TCAACAGCAGCGATGACAAT
soxR	948566	243	AATCAGCGGCGATATAAACG	AAGGCAGCCACAACCAATAC
sulA	947335	175	TGGTTAACACCGCAACAAAA	CGCATCAACAAGTTCAGCAT

Supplementary Table 3 PNA structures

PNA name	Sequence	Target	Target location and length (10 nucleotides)	Reference
PNA-*FtsZ*	(KFF)3K-eg1-tcaaacatag	*FtsZ*	-2 to +8	[12]
PNA-*FabI*	(KFF)3K-eg1-cccatagctt	*FabI*	-5 to +5	[13]

References

[1] Simons, R., Houman, F. & Kleckner, N. Improved single and multicopy lac-based cloning vectors for protein and operon fusions. *Gene* **53**, 85−96 (1987).

[2] Kobayashi, D. *et al.* Endogenous reactive oxygen species is an important mediator of miconazole antifungal effect. *Antimicrob Agents Chemother* **46**, 3113−3117 (2002).

[3] Oberto, J., Nabti, S., Jooste, V., Mignot, H. & Rouviere-Yaniv, J. The HU regulon is composed of genes responding to anaerobiosis, acid stress, high osmolarity and SOS induction. *PLoS One* **4**, e4367 (2009).

[4] Miller, J. H. in *Experiments in Molecular Genetics* (ed Jeffrey H. Miller) Ch. 48, 352−355 (Cold Spring Harbor Laboratory, 1972).

[5] Griffith, K. L. & Wolf, R. E. Measuring β-galactosidase activity in bacteria: cell growth, permeabilization, and enzyme assays in 96-well arrays. *Biochem Biophys Res Commun* **290**, 397−402 (2002).

[6] Livak, K. J. & Schmittgen, T. D. Analysis of relative gene expression data using real-time quantitative PCR and the $2^{-\Delta\Delta C_T}$ Method. *Methods* **25**, 402−408 (2001).

[7] Datsenko, K. A. & Wanner, B. L. One-step inactivation of chromosomal genes in *Escherichia coli* K-12 using PCR products. *Proc Natl Acad Sci U S A* **97**, 6640−6645 (2000).

[8] Coulondre, C. & Miller, J. Genetic studies of the lac repressor. III. Additional correlation of mutational sites with specific amino acid residues. *J Mol Biol* **117**, 525−567 (1977).

[9] Sekiguchi, M. & Iida, S. Mutants of *Escherichia coli* permeable to actinomycin. *Proc Natl Acad Sci U S A* **58**, 2315−2320 (1967).

[10] Baba, T. *et al.* Construction of *Escherichia coli* K-12 in-frame, single-gene knockout

mutants: the Keio collection. *Mol Syst Biol* **2**, 1−11 (2006).

[11] Kitagawa, M. *et al.* Complete set of ORF clones of *Escherichia coli* ASKA library (a complete set of *E. coli* K-12 ORF archive): unique resources for biological research. *DNA Res* **12**, 291−299 (2006).

[12] Goh, S., Boberek, J. M., Nakashima, N., Stach, J. & Good, L. Concurrent growth rate and transcript analyses reveal essential gene stringency in *Escherichia coli*. *PLoS One* **4**, e6061 (2009).

[13] Dryselius, R., Nekhotiaeva, N. & Good, L. Antimicrobial synergy between mRNA-and protein-level inhibitors. *J Antimicrob Chemother* **56**, 97−103 (2005).
